# Pro- and anti-inflammatory responses of peripheral blood mononuclear cells induced by *Staphylococcus aureus* and *Pseudomonas aeruginosa* phages

**DOI:** 10.1038/s41598-017-08336-9

**Published:** 2017-08-14

**Authors:** Jonas D. Van Belleghem, Frédéric Clement, Maya Merabishvili, Rob Lavigne, Mario Vaneechoutte

**Affiliations:** 1 Laboratory Bacteriology Research, Department of Clinical Chemistry, Microbiology and Immunology, University Ghent, Medical Research Building II, De Pintelaan 185, 9000 Ghent, Belgium; 2 Center for Vaccinology, Ghent University Hospital, Ghent, Belgium; 3 Laboratory for Molecular and Cellular Technology (LabMCT) Queen Astrid Military Hospital, Bruynstraat 1, 1120 Brussels, Belgium; 4 Laboratory of Gene Technology, KULeuven, Kasteelpark Arenberg 21 box 2462, 3001 Leuven, Belgium

## Abstract

The ability of bacteriophages to kill bacteria is well known, as is their potential use as alternatives to antibiotics. As such, bacteriophages reach high doses locally through infection of their bacterial host in the human body. In this study we assessed the gene expression profile of peripheral blood monocytes from six donors for twelve immunity-related genes (*i*.*e*. *CD14*, *CXCL1*, *CXCL5*, *IL1A*, *IL1B*, *IL1RN*, *IL6*, *IL10*, *LYZ*, *SOCS3*, *TGFBI* and *TNFA*) induced by *Staphylococcus aureus* phage ISP and four *Pseudomonas aeruginosa* phages (*i*.*e*. PNM, LUZ19, 14-1 and GE-vB_Pae-Kakheti25). The phages were able to induce clear and reproducible immune responses. Moreover, the overall immune response was very comparable for all five phages: down-regulation of *LYZ and TGFBI*, and up-regulation of *CXCL1*, *CXCL5*, *IL1A*, *IL1B*, *IL1RN*, *IL6*, *SOCS3* and *TNFA*. The observed immune response was shown to be endotoxin-independent and predominantly anti-inflammatory. Addition of endotoxins to the highly purified phages did not cause an immune response comparable to the one induced by the (endotoxin containing) phage lysate. In addition, the use of an intermediate level of endotoxins tipped the immune response to a more anti-inflammatory response, *i*.*e*. up-regulation of IL1RN and a strongly reduced expression of CXCL1 and CXCL5.

## Introduction

Bacteriophages are the most abundant entities, impacting ecological niches ranging from the ocean to the gut microbiome^1–5^. Furthermore, it is known that phages are immensely specific towards a specific bacterial host without infect other bacterial strains. Moreover, there is currently no data available of phages infecting eukaryotic cells. This makes them ideal candidates to treat bacterial infections, while being harmless to mammalian cells and even non-target bacteria^6^.

Bacteria that inhabit the intestine and skin are generally regarded as stable residents that may confer metabolic and/or immune benefits to their hosts^7^. The host immune system has evolved mechanisms to tolerate these commensal organisms while at the same time providing protection for the host from pathogens^8^. Similarly, metagenomic studies have revealed that a vast variety of bacteriophages are associated with healthy human tissues^9–11^. In case of phages, a persistent nonpathogenic association seems possible as viral replication occurs only in bacterial hosts, which can themselves be stable members of the microbiome^12^.

It has been demonstrated that oral uptake of phages by animals results in the translocation of phages to systemic tissues^13, 14^. This suggests that mammals have mechanisms for the uptake and delivery of phages from the gut to the blood. The contact between systemic tissues and phages may allow intestinal phages to elicit innate and adaptive immune responses. One possible uptake route involves dendritic cells, which are known to sample intestinal luminal contents and can actively phagocytize phage particles in culture^15, 16^. It is also interesting to consider whether phages might elicit antiviral innate immune responses. Mammalian cells are endowed with the ability to detect viral nucleic acids through several pattern-recognition receptors that are positioned to detect viral entry into cells^12^.

A study performed by Weber-Dabrowska *et al*.^17^ demonstrated that phage therapy can normalize TNF-α serum levels and the production of TNF-α and IL6 by blood cell cultures. Miernikiewicz *et al*.^18^) performed an extensive study of the immunological effects of phage T4 and its head surface proteins. They found that phage T4 and its surface proteins gp23*, gp24*, Hoc and Soc did not affect production of the inflammatory cytokine and ROS production. Recently Majewska *et al*.^19^ followed the antibody production (*i*.*e*. IgM, IgG and secretory IgA) after oral application of phage T4 to mice. However, the orally applied phage T4 induced anti-phage antibodies only after a combination of long exposure times (*i*.*e*. IgG day 36 and IgA day 79) and high doses.

The use of phage therapy has persisted without interruption in Eastern Europe, particularly in centers such as the Eliava Institute of Bacteriophage, Microbiology and Virology in Tbilisi, Georgia and the Institute of Immunology and Experimental Therapy in Wroclaw, Poland^20, 21^. Phage interactions with animals in general and human beings in particular have been comprehensively reviewed^21^, and there have been no reports of significant adverse reactions despite their long history of administration to humans.

Here, we report on the immune response induced by individual (highly purified) phages. It has previously been described that CsCl gradient ultra-centrifuged phages are free from residual DNA, RNA and bacterial proteins released during the lysis of the bacterial cell^22^. Hence the immune responses observed in our study are induced by the phages. We conducted a whole transcriptome analysis of human peripheral blood mononuclear cells (PBMCs) stimulated with either *P*. *aeruginosa* or a *P*. *aeruginosa* phage PNM lysate. The *P*. *aeruginosa* phage PNM lysate condition most closely reflects the immunological condition obtained during phage therapy, when the phage titer is the highest and predominantly bacterial fragments, from the lysed bacterial cells, remain. These data suggest that certain immunological pathways are activated during and/or after phage therapy and may contribute to the efficacy of phages during phage therapy. Next, to understand to what extent the phage particles interact with the human immune system, the immune response induced by two different phage purification strategies (*i*.*e*. either a phage lysate or a highly purified phage) was compared. Using twelve immunity-related genes, the immune response induced by five different phages were compared, four infecting *P*. *aeruginosa* and one *S*. *aureus* phage. These five phages represent the three major phage morphologies (Table 1). This might give us an idea whether the immune response might be phage host specific or phage morphology dependent.

**Table 1:** Different phages used in the different purification strategies.

Bacteriophage	Phage family	Bacterial Host	Titer (pfu/ml)		Endotoxins (EU/ml)		Reference	Isolated by	Isolation date
			Lysate	Higly purified	Lysate	Higly purified			
*P*. *aeruginosa* phage PNM	*Podoviridae*	*P*. *aeruginosa* strain 573	1.8 × 10^13^	3.0 × 10^13^	3.1 × 10^6^	6.6 × 10^3^	Merabishvili *et al*.^54^	N. Lashki & M. Tediashvili	1999
*P*. *aeruginosa* phage LUZ19	*Podoviridae*	*P*. *aeruginosa* strain 573	5.0 × 10^13^	5.0 × 10^11^	3.1 × 10^4^	4.2 × 10^3^	Lammens *et al*.^66^	P. J. Ceyssens	2006
*P*. *aeruginosa* phage GE-vB_Pae-Kakheti25	*Siphoviridae*	*P*. *aeruginosa* strain 573	2.5 × 10^12^	9.0 × 10^10^	7.5 × 10^6^	1.3 × 10^3^	Karumidze *et al*.^67^	N. Kvatadze	2012
*P*. *aeruginosa* phage 14-1	*Myoviridae*	*P*. *aeruginosa* strain 573	3.6 × 10^12^	5.0 × 10^12^	3.3 × 10^5^	3.5 × 10^4^	Ceyssens *et al*.^68^	V. Krylov	2000
*S*. *aureus* phage ISP	*Myoviridae*	*S*. *aureus* strain ATCC 6538	2.0 × 10^11^	1.4 × 10^11^	0.0	0.0	Vandersteegen *et al*.^69^	Unknown	1920–1930

For each phage, taxonomical family and bacterial host strain are presented, as well as the titer of the phage lysates, before and after endotoxin removal.

## Results

### RNA transcriptome analysis of peripheral blood mononuclear cells

Peripheral blood mononuclear cells (PBMCs) derived from one donor were stimulated in triplicate with either saline, 0.1 cfu/PBMC of *P*. *aeruginosa* strain 573 or 10^3^ pfu/PBMC of *P*. *aeruginosa* phage PNM, during 20 h. An average of 47 million reads was obtained for the unstimulated and stimulated PBMCs, after cleaning and quality checks were carried out. Of the total number of reads, approximately 85% could be mapped to the human genome (Table S1).

Transcriptome analysis of the unstimulated PBMCs versus *P*. *aeruginosa* strain 573 revealed a total of 996 up-regulated genes and 1377 down-regulated genes compared to 704 up-regulated and 392 down-regulated genes when the PBMCs were stimulated with *P*. *aeruginosa* phage PNM (Tables S2 and S3). Many of the genes that are differentially expressed play important roles in immunity-related processes. For example, the Major Histocompatibility Class (MHC) II genes HLA-DMA, HLA-DMB, HLA-DRB1 and HLA-DRB4 were significantly down-regulated after phage PNM stimulation. In case of PBMCs stimulated with *P*. *aeruginosa*, our results suggest that most of the genes involved in the antigen presentation by MHC I and MHC II are not differentially expressed, with exception of HLA-DRB4 (Log2 fold change of −1.00) and HLA-L (Log2 fold change of 1.02). CD1c is also significantly down-regulated in the *P*. *aeruginosa* phage PNM dataset. The CD1 molecules are MHC-like proteins that bind β2 microglobulin but, in contrast to MHC class I, their principal domain of action is in the endocytic pathway^23^.

An observation that might play a crucial role in the immune response against the bacterial host of phage PNM, *i*.*e*. *P*. *aeruginosa*, is the down-regulation of Toll-like receptor 4 (TLR4). Toll-like receptor 4 is a classic member of the TLR superfamily and has been studied extensively in pathogen-mediated host responses and functions as a primary sensor to detect LPS, a component of Gram-negative bacteria such as *P*. *aeruginosa* ^24^. TLR4 activation induces secretion of pro-inflammatory molecules such as chemokines and cytokines which amplify the response to infection^25, 26^.

Both *P*. *aeruginosa* and phage PNM have the ability to down-regulate TLR4, a −3.9 Log2 fold change reduction and −1.3 Log2 fold change reduction, respectively. In addition, *P*. *aeruginosa* was able to down-regulate CD14 (Log2 fold change of −8.0) and lymphocyte antigen 96, encoded by the *LY96* gene (Log2 fold change of −2.0), also known as MD2. Both genes play an important role as co-receptors of TLR4 in the recognition of Gram-negative LPS. Furthermore, it is interesting to note that lysozyme is strongly down-regulated after *P*. *aeruginosa* strain 573 stimulation and *P*. *aeruginosa* phage PNM (Log2 fold change of −7.8 and −3.3, respectively).

Besides the differential expression in the MHC class II genes, many cytokines were up- and down-regulated. Of the differentially expressed cytokines, the IL10 family cytokines (*i*.*e*. IL-10, IL-19, IL-20, IL-22, IL-24, IL-26, IL-28A and IL-28B) are of particular interest for their anti-inflammatory properties and tissue protection^27^. IL-10, known for its interaction with leukocytes and its anti-inflammatory properties, had an up-regulation after phage PNM stimulation (Log2 fold change of 4.3), and after *P*. *aeruginosa* stimulation (Log2 ratio of 4.3).

IL-10 is an anti-inflammatory cytokine that inhibits the activity of T_H_1 cells, NK cells and macrophages^28^. It blocks the expression of pro-inflammatory cytokines, including TNF-α, IFN-γ, IL-1, IL-12, and CXC and CC chemokines. The suppression of these cytokines is clearly visible in our data, (Tables S2 and S3). It also suppresses MHC class II and co-stimulatory molecules CD80 and CD86 on macrophages, and it inhibits the generation of reactive oxygen and nitrogen intermediates from macrophages and neutrophils. Our data indicate a slight up-regulation of CD80 (Log2 ratio of 1.7 for phage PNM and Log2 fold change of 1.6 for *P*. *aeruginosa*) and a down-regulation of CD86 (Log2 ratio of −1.5 for phage PNM and Log2 fold change of −1.8 for *P*. *aeruginosa*).

The IL-20 subfamily cytokines, composed of IL-19, IL-20, IL-24 and IL-26, primarily acts on various epithelial cells and protects these cells from invasion by extracellular pathogens such as bacteria and yeast. Of these, IL-19 (Log2 ratio of 3.1 for phage PNM and Log2 ratio of 2.77 for *P*. *aeruginosa*), IL-20 (Log2 ratio of 7.1 for phage PNM and Log2 ratio of 5.7 for *P*. *aeruginosa*) and IL-24 (Log2 ratio of 3.2 for phage PNM and Log2 ratio of 1.1 for *P*. *aeruginosa*) were differently expressed.

Interleukin-6 (IL-6, Log2 ratio of 6.6 for phage PNM, Log2 fold change of 6.4 for *P*. *aeruginosa*), originally considered to be a pro-inflammatory cytokine, has anti-inflammatory properties as well^29^. For instance, IL-6 inhibits neutrophil accumulation after lipopolysaccharide (LPS) injection and antagonizes the actions of interleukin-1β (IL-1β) and tumor necrosis factor-α (TNF-α) via induction of the soluble IL-1 receptor antagonist and the soluble TNF-α receptor^30^.

Suppressors of cytokine signaling (SOCS) protein are a family of eight intracellular proteins that control cytokine signaling by suppressing cytokine signal transduction process, *i*.*e*. SOCS-1 to -7 and cytokine-inducible SH2-containing protein (CIS). SOCS-1 and -3 specifically participate in regulation of Th1 and Th2 cytokine signaling^31, 32^. SOCS-1 (Log2 fold change of 1.9 for phage PNM and Log2 ratio of 1.4 for *P*. *aeruginosa*) is a key modulator of interferon-γ (IFN-γ) signaling. Mice lacking SOCS-1 exhibit deregulated responses to IFN-γ resulting in excessive T-cell activation and are hyper-responsive to viral infections^33, 34^. SOCS-3 (Log2 fold change of 2.0, no differential expression for *P*. *aeruginosa*) functions to control IL-6 induced Th2-associated response via its receptor gp130^35^. In macrophages, SOCS-3 mediates IL-10 inhibition of TNF-α and nitric oxide production^36^. Induction of SOCS-3 is regulated via IL-6 receptor trans-signaling. The observation of increased IL-10 in SOCS-3 over-expressed trophoblast after IL-6 challenge is in line with the concept of a pro-inflammatory role of IL-6 in trophoblasts and SOCS-3 signaling in regulation with cytokine production^37^.

When comparing both the *P*. *aeruginosa* strain 573 and *P*. *aeruginosa* phage PNM lysate data sets, 359 differentially expressed genes could be identified which were exclusively present in the phage PNM stimulated dataset. Of these 359 differentially expressed genes, 117 were up-regulated whereas 242 were down-regulated (Table S4). Therefore, expression of these genes can be considered as uniquely caused by the phage, and not by contamination of the phage with bacterial antigens. To further deduce whether the phage has immune properties, RT-qPCR analysis was performed on RNA derived from the PBMCs of six donors, stimulated during 20 hours with different concentrations of phage lysates, endotoxin-purified phages (Table 1), bacterial host strains and/or endotoxins, for a selection of twelve immunity-related genes (Table S5; discussed below).

### RT-qPCR validation of 12 immunity-related genes

#### *Staphylococcus aureus* phage ISP induced immune response

**Figure 1:**
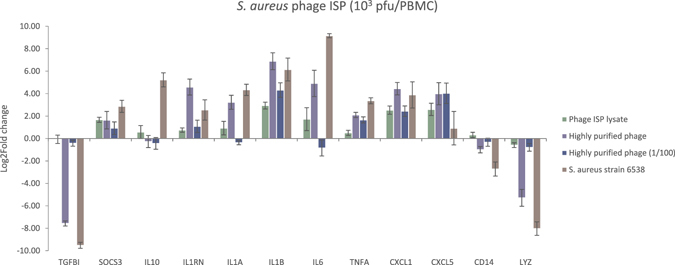
Gene expression analysis of 12 immunity-related genes, assessed by means of RT-qPCR, after 20 h stimulation of PBMCs with *S*. *aureus* strain ATCC 6538 or *S*. *aureus* phage ISP. Comparison of the immune response of the PBMCs, expressed as log2 fold change, after normalization with reference gene ACTB, as induced by the phage lysate, highly purified phage (undiluted and diluted hundredfold) or its bacterial host (*i*.*e*. *S*. aureus strain ATCC 6538). The immune response induced by the highly purified phage preparation is stronger than the one induced by the phage lysate. Diluting of the highly purified phage led to an almost complete reduction of the observed immune response, with the exception of *CXCL5* and *IL1B*. A strong difference is present between the immune response induced by the phage compared to its bacterial host (*i*.*e*. *S*. *aureus* strain 6538).

Stimulation of PBMCs, derived from six donors (each stimulated in triplicate), with either an *S*. *aureus* phage ISP lysate, the highly purified phage ISP (undiluted and 100-fold diluted), or the *S*. *aureus* host strain showed that the immune response within each condition was highly reproducible (Fig. 1). Diluting the highly purified phage ISP led to an almost complete reduction of the observed immune response, with the exception of *TNFA* and *CXCL5* which have the same induction as the undiluted phage and *IL1B* and *CXCL1* which are both up-regulated but are significantly different from the undiluted phage condition, *i*.*e*. up-regulated but significantly less strong than the undiluted phage. These clear titration effects indicate that sufficiently high phage titers (*i*.*e*. ≥10^3^ pfu/PBMC) are necessary in order to induce an immune response.

Interestingly, the highly purified phage ISP induced an almost identical response as its bacterial host strain (except for *CXCL5*, *IL1RN*, *IL6 and IL10*), whereas the phage lysate, still containing bacterial contamination, induced a much weaker overall response (except for *CXCL1*, *CXCL5*, *IL1B*, *IL1RN* and *TNFA*).

The highly purified phage ISP significantly (p-value < 0.05) up-regulates the *CXCL1*, *IL1A*, *IL1B*, *IL1RN*, *IL6* and *TNFA* genes and down-regulates *CD14*, *LYZ* and *TGFBI* compared to the phage lysate.

The immune response induced by the phage and its bacterial host (*i*.*e*. *S*. *aureus* strain 6538) also shows a strong down-regulation of the *LYZ* gene expression, in agreement with the transcriptome results (Fig. 1). The values between the transcriptome analyses and RT-qPCR were consistent (R² = 0.88).

#### Influence of endotoxins on the phage-induced immune response

To determine whether the observed immune response was induced by the bacteriophage rather than by possible endotoxin contamination present in the phage lysate or purified phage (Table 1), PBMCs were stimulated with either *S*. *aureus* phage ISP alone, *i*.*e*. completely endotoxin free (Table 1), at concentrations of 10^3^ pfu/PBMC, or in combination with different concentrations of a commercial endotoxin preparation (*i*.*e*. 0.1, 10^−3^ or 10^−7^ EU/PBMC).

**Figure 2:**
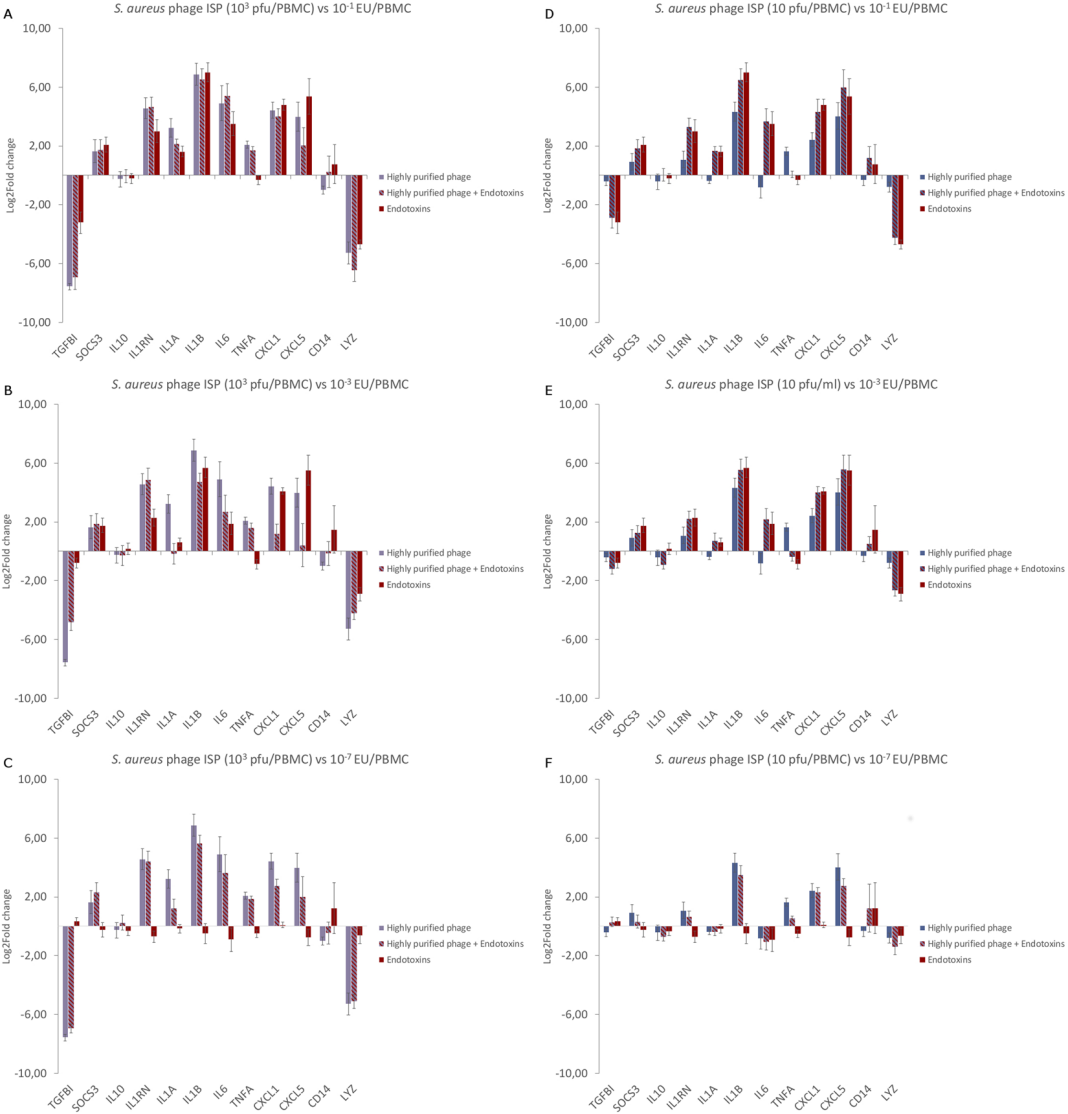
Gene expression analysis of 12 immunity-related genes, assessed by means of RT-qPCR, after 20 h of stimulation of PBMCs with *S*. *aureus* phage ISP in combination with endotoxins (*i*.*e*. 0.1, 10^−3^ or 10^−7^ EU/ml). (**A**,**B**,**C**) 10^3^ pfu/PBMC in combination with (**A**) 0.1 EU/PBMC, (**B**) 10^−3^ EU/PBMC or (**C**) 10^−7^ EU/PBMC leads to an immune response more similar to the one induced by the phage alone. (**D**,**E**,**F**) Reducing the phage titer to 10 pfu/PBMC causes the immune response to tilt in the direction of the endotoxin induced immune response instead of the phage induced immune response.

The addition of up to 0.1 EU/PBMC to 10^3^ pfu/PBMC of phage ISP did not lead to a significant difference in the immune response as induced by the phage alone (Figs 2A–C and S1–S3). However, the immune response induced by phage ISP at 10^3^ pfu/PBMC (with or without endotoxins) differed significantly (p-value < 0.05) from the immune response induced by the endotoxins alone, for *CXCL5*, *LYZ*, *TGFBI* and *TNFA*. The combination of phage with endotoxins led to a stronger up-regulation of *TNFA* or stronger down-regulation of *LYZ* and *TGFBI* in comparison with endotoxins alone, whereas the expression of *CXCL5* was reduced compared to endotoxin alone when a combination of phage ISP with endotoxins was used.

Lowering the endotoxin concentration added to phage ISP from 0.1 EU/PBMC to 10^−3^ EU/PBMC, resulted in a significant difference (p-value < 0.05) in the gene expression of *CXCL1*, *CXCL5*, *IL1RN*, *LYZ*, *TNFA* and *TGFBI*, between the phage ISP with endotoxins (*i*.*e*. 10^3^ pfu and 10^−3^ EU/PBMC) and endotoxins alone (*i*.*e*. 10^−3^ EU/PBMC). Of particular interest was the down-regulation of *CXCL1* and *CXCL5* when stimulation was carried out with both endotoxin and phage compared to 10^3^ pfu of phage ISP/PBMC alone or 10^−3^ of EU/PBMC alone (Figs 2B and S2). Further reduction of the endotoxin concentration to 10^−7^ EU/PBMC did not significantly differ from the immune response induced by the phage alone, with the exception of *CXCL1* and *IL1A* (Figs 2C and S3), which were less expressed when the PBMCs were challenged with 10^−7^ EU/PMBC added to the phage instead of when stimulated by the phage alone.

Lowering the phage concentration from 10^3^ pfu/PBMC to 10 pfu/PBMC, in combination with 0.1, 10^−3^ or 10^−7^ EU/PBMC led to an immune response more similar to the one induced by the endotoxin alone than the one induced by the phage alone (Fig. 2D–F).

#### *P. aeruginosa* phage PNM induced immune response

**Figure 3:**
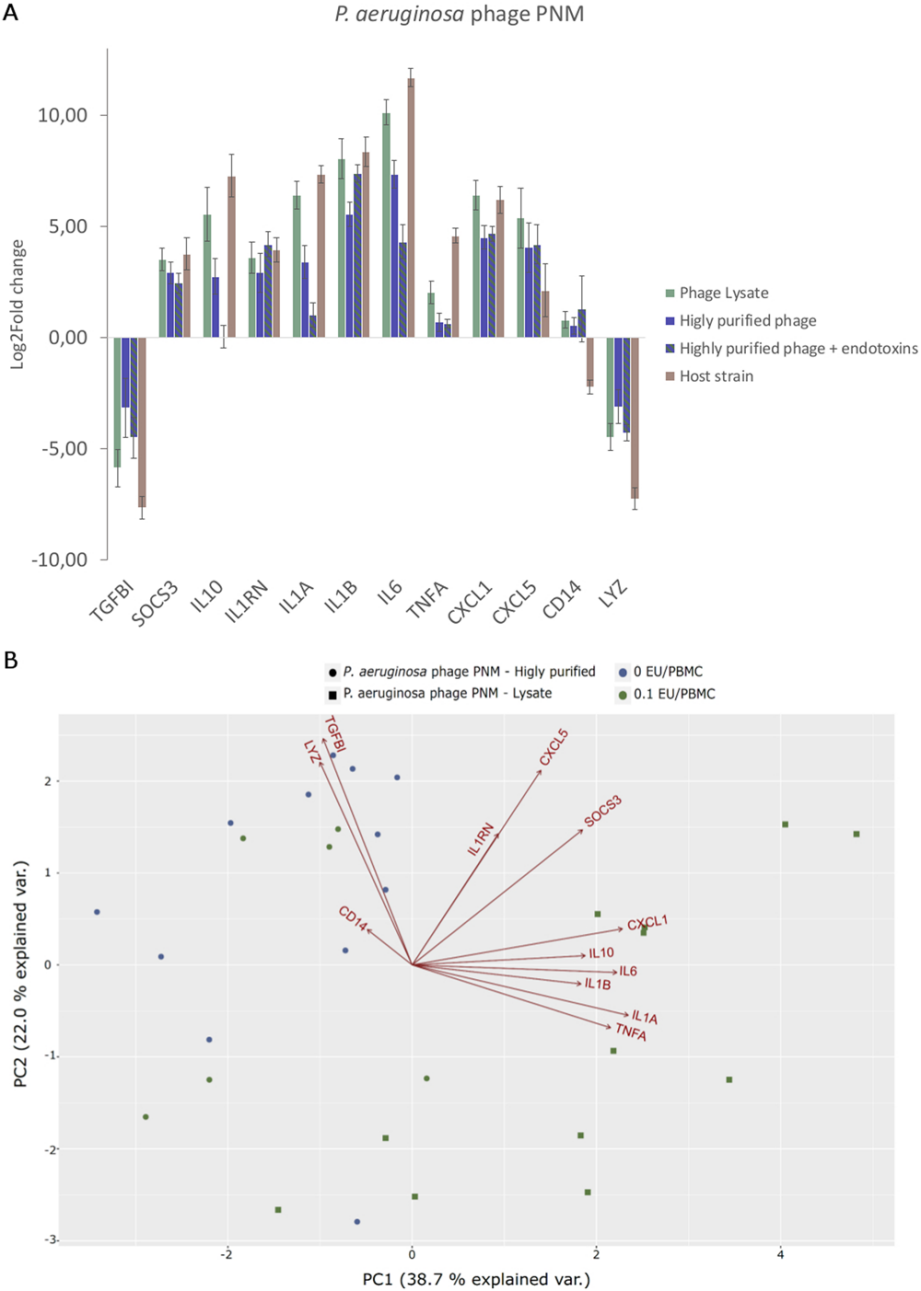
Effect of endotoxins in the presence *P. aeruginosa* phage PNM. (**A**) Gene expression analysis of 12 immunity-related genes by means of RT-qPCR after 20 h of stimulation of PBMCs. PBMCs were stimulated with *P*. *aeruginosa* phage PNM, either a phage lysate (10^5^ pfu/PBMC; 0.1 EU/PBMC) or a highly purified phage preparation (10^5^ pfu/PBMC; 10^−5^ EU/PBMC) in combination with 0.1 EU/PBMC. The pro-inflammatory markers *IL1A*, *IL1B*, *CXCL1* and *CXCL5*, and the anti-inflammatory markers SOCS3, IL10, *IL1RN* and *IL6* are upregulated The addition of 0.1 EU/PBMC to the highly purified *P*. *aeruginosa* phage preparation does not revert the observed immune response to that of the phage lysate for *IL10*, *IL1A*, *IL6*, *TNFA* and *CXCL1*. (**B**) Principal components analysis of *P*. *aeruginosa* phage PNM with or without the addition of endotoxins. The immune response induced by the highly purified phage PNM () differs from the one induced by the phage PNM lysate (), as these two groups are visibly separated. When endotoxins are added to a final concentration of 0.1 EU/PBMC to the highly purified phage (), the immune response is similar to the highly purified phage () and not towards the phage lysate (), indicating that the observed difference is not due to the presence of LPS but due to bacterial proteins present in the phage lysate.

Comparable to the observations for *S*. *aureus* phage ISP, the immune response induced by the *P*. *aeruginosa* phage PNM lysate (at 10^5^ pfu/PBMC) differs significantly (p-value < 0.05) from the one induced by the highly purified phage (at 10^5^ pfu/PBMC). However, it was the *P*. *aeruginosa* phage PNM lysate-induced response that was most consistent with the response to its bacterial host strain (*i*.*e*. 0.1 cfu/ml), except for *CD14*, *CXCL5*, *LYZ* and *TNFA*. A significant difference in the induced immune response between the phage lysate and the purified phage could be observed for *CXCL1*, *IL1A*, *IL1B*, *IL6*, *TNFA* and *TGFBI* (Fig. 3A). Because this difference could be due to endotoxin contamination, we compared the stimulation of the PBMCs with a highly purified phage PNM preparation with the addition of endotoxins (*i*.*e*. 0.1 EU/PBMC), in order to equalize the endotoxin concentration with that of the phage lysate (0.3 EU/PBMC, Table 1). Nonetheless, this did not bring the observed immune response closer to the one induced by the phage lysate and even had the opposite effect for *e*.*g*. *IL1A*, *IL6* and *IL10* (Fig. 3A and B). This indicates that the difference in immune response induced by the highly purified phage compared to the phage lysate is not due to the presence of endotoxins in the phage lysate, but rather due to the presence of bacterial contaminants (*e*.*g*. bacterial DNA or proteins) in the phage lysate.

#### Immune response induced by *P. aeruginosa* phages PNM, LUZ19, 14-1 and GE_vB_Pae-Kakheti25

**Figure 4:**
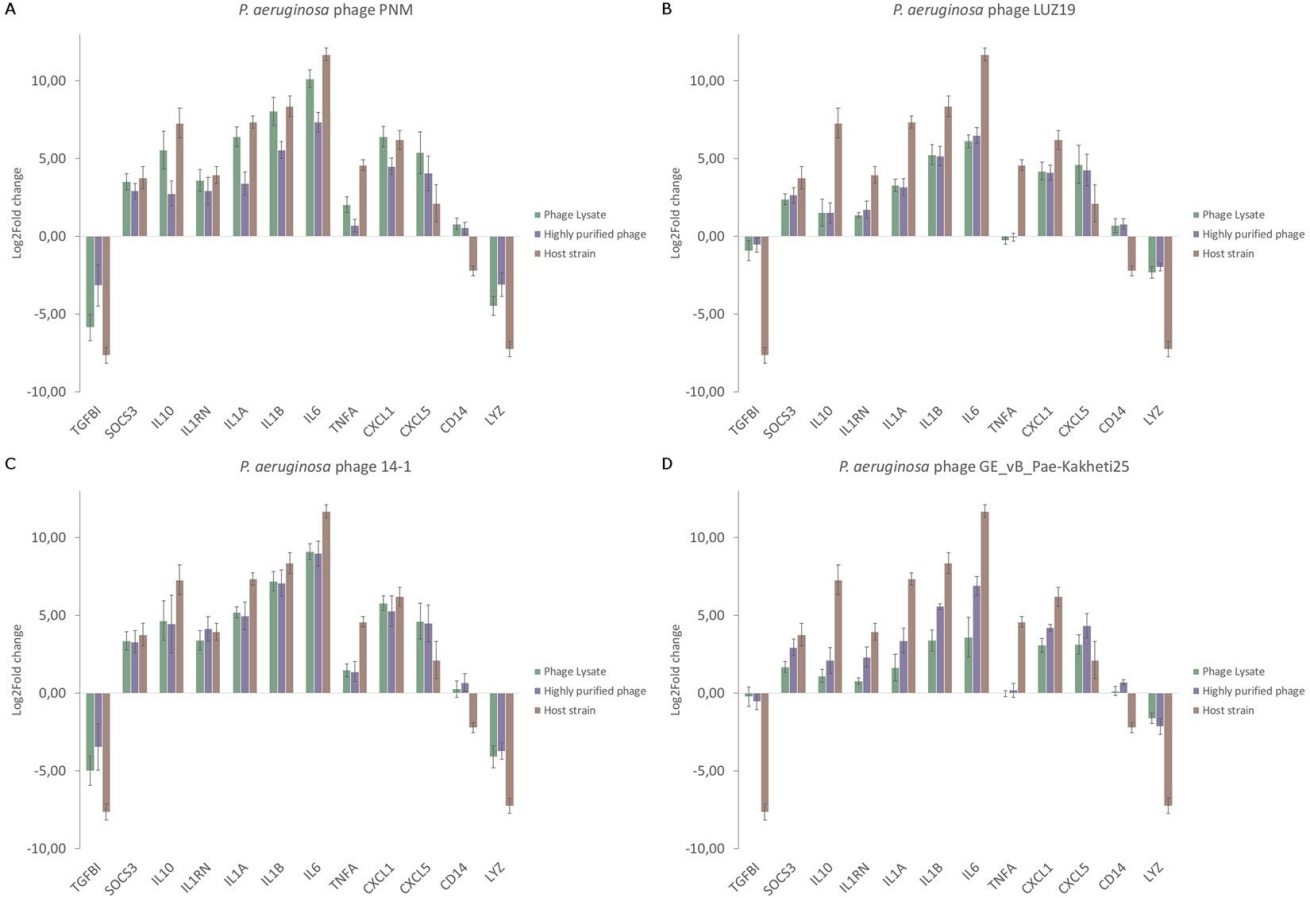
Gene expression analysis of 12 immunity-related genes by means of RT-qPCR after 20 h stimulation of PBMCs. PBMCs were stimulated with either (**A**) *P*. *aeruginosa* phage PNM, (**B**) *P*. *aeruginosa* phage LUZ19, (**C**) *P*. *aeruginosa* phage 14-1 or (**D**) *P*. *aeruginosa* phage GE_vB_Pae-Kakheti25. The pro-inflammatory markers *IL1A*, *IL1B*, *CXCL1* and *CXCL5* and the anti-inflammatory markers *SOCS3*, *IL10*, *IL1RN* and *IL6* are up-regulated. Only phages PNM **(A)** and 14-1 (**C**) slightly up-regulate *TNFA*, whereas phages LUZ19 (**B**) and GE_vB_Pae-Kakheti25 (**D**) cause a slight down-regulation of *TNFA*.

All four *P*. *aeruginosa* phages [*i*.*e*. PNM (10^5^ pfu/PBMC), LUZ19 (10^5^–10^3^ pfu/PBMC), 14-1 (10^4^–10^3^ pfu/PBMC and Ge_vB_Pae-Kakheti25 (10^4^ pfu/PBMC)] induced a comparable immune response (Fig. 4). For all four phages, there is a clear difference in the level of the gene expression induced in the PBMCs by the stimulation of the phage lysate or the highly purified phage. For all four phages, the phage lysate induces a stronger immune response compared to the highly purified phage.

All four *P*. *aeruginosa* phages induce IL6 and the anti-inflammatory genes *IL1RN*, *IL10* and *SOCS3*, as well as the pro-inflammatory genes *CXCL1*, *CXCL5*, *IL1A* and *IL1B*. Only the expression of *TNFA* is different between the four phages, with phage PNM and 14-1 inducing a significant slight up-regulation while phages LUZ19 and GE_vB_Pae-Kakheti25 induced a down-regulation (Fig. 4).

Moreover, the phage lysates strongly resemble the immune response induced by their bacterial host, *i*.*e*. *P*. *aeruginosa* strain 573.

## Discussion

Transcriptome analysis of PBMCs stimulated with *P*. *aeruginosa* phage PNM showed the differential expression of different immunity-related genes. Subsequent in depth analysis of twelve selected immunity-related genes (Table S5) by means of RT-qPCR confirmed that five different bacteriophages, one with a Gram-positive host and four with a Gram-negative host (Table 1), were able to induce an immune response. Our study addressed only the cytokine gene expression level and not the cytokine protein level, for several reasons. First, stimulation times for cytokine protein levels are much longer^38, 39^ and such longer stimulation times are not optimal for freshly isolated PBMCs. Furthermore, an early response and fluctuations in the response are more rapidly detected at the gene transcription level than at changes in protein concentration.

**Figure 5:**
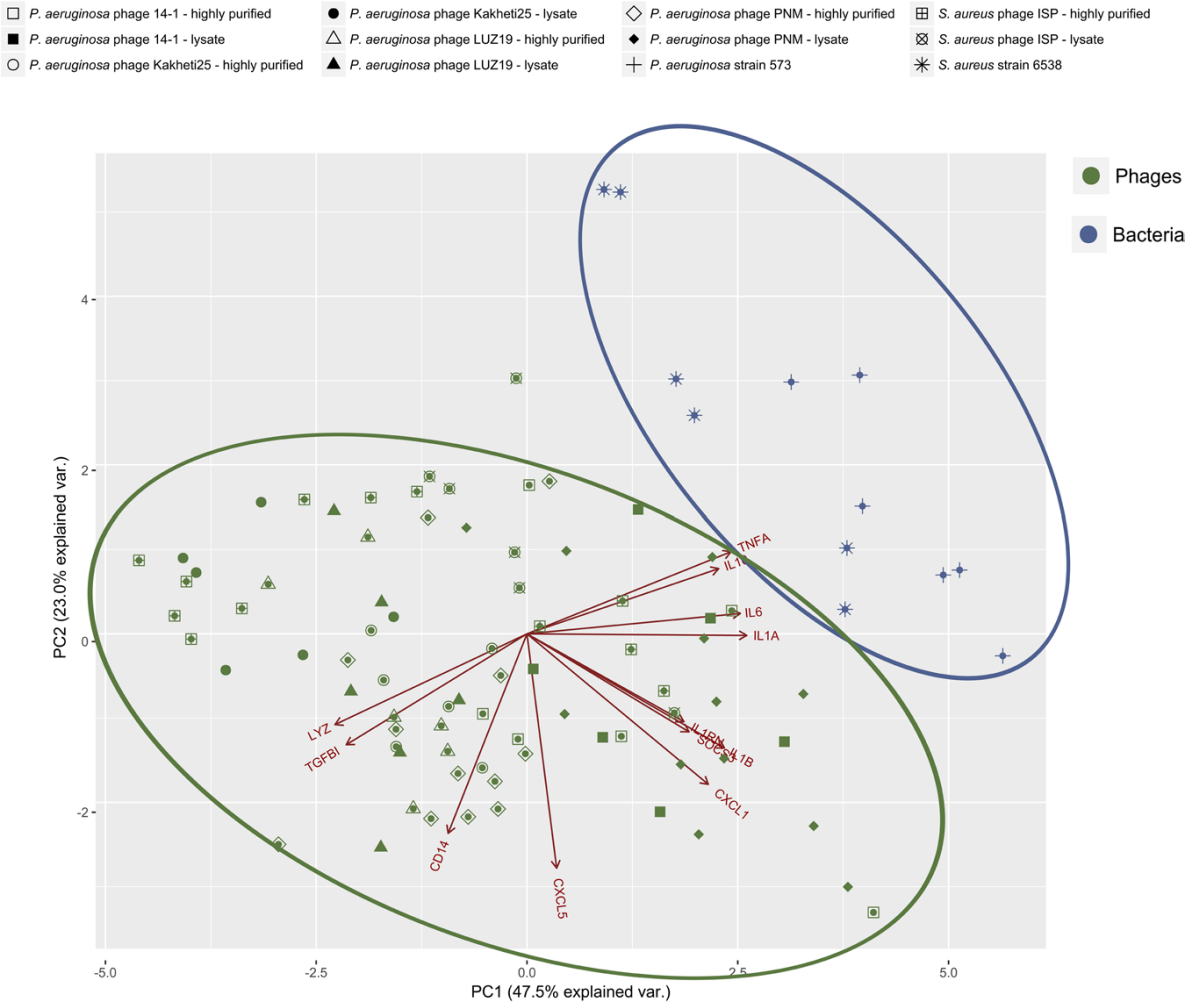
Principal component analysis (PCA). Comparison of the immune response of PBMCs induced by all five tested phages (Highly purified or phage lysate; magenta) and their bacterial host strain (blue). The PCA clearly shows that the bacteria (blue) and phages (magenta) for two separate groups, indicating that the immune response induced is different between these two types of stimuli. There is no clear distinction between the different phages, indicating that these five different phages induced similar responses. Moreover the gene expression of *IL1RN*, *IL1B* and *SOCS3*, *IL6* and *IL1A*, *TNFA* and *IL10*, and *LYZ* and *TGFBI* are correlated.

We found that for all five phages, the overall immune response (as determined by RT-qPCR) is very comparable (Fig. 5), but differs from the immune response induced by their bacterial host. The observed immune response is largely in correspondence with the transcriptome analysis. Moreover, the immune response induced by a large number of phages (*i*.*e*. 10^3^ pfu/PBMC) seems to be endotoxin independent; the addition of endotoxins to the highly purified *S*. *aureus* phage ISP did not subvert the immune response to the one induced by the *S*. *aureus* phage ISP lysate. Furthermore, the stimulation of PBMCs with either a *P*. *aeruginosa* phage PNM lysate or a highly purified *P*. *aeruginosa* phage PNM preparation supplemented with endotoxins in concentration of 0.1 EU/PBMC (Fig. 3) did not bring the observed immune response closer to the one induced by the phage PNM lysate, indicating that the immune response induced by the phage preparations is not due to the presence of endotoxins but more due to presence of other bacterial components in the phage lysate preparation. Additionally, it has recently been shown that *Escherichia coli* phage T4 gp12 can decrease the immune response induced by endotoxins, through the binding of endotoxins to gp12^40^.

Whether phages induce a highly comparable overall immune response or whether only specific parts of the immune response are similar needs to be further evaluated, as we specifically looked at twelve immunity-related genes. However, the ability of these five phages to induce a, largely, similar immune response might be due to the modular nature of phage genomes^41^. Based on the modular nature of phages genomes, it can be hypothesized that phage capsid proteins might have similar folds and subsequently induce similar immune responses. For instance, it has been shown that gp23 and gp24 of *E*. *coli* phage T4 have a similar fold as that of the *E*. *coli* phage HK97 capsid protein^42, 43^. Yet a single point mutation can change the serotype of the phage. This might also be the case for *P*. *aeruginosa* phage PNM and LUZ19, which are homologous, yet slightly differ in the induced immune response.

**Figure 6:**
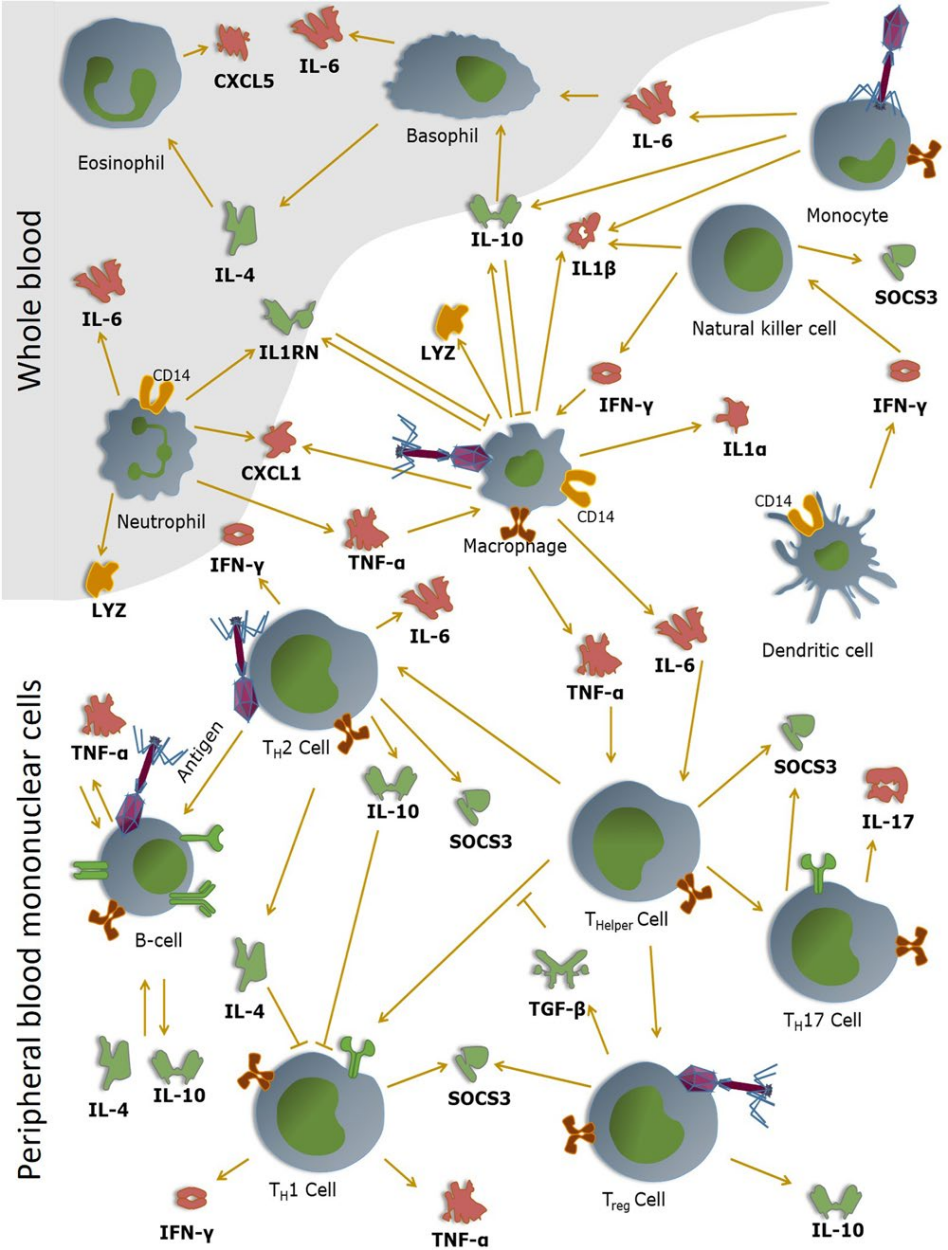
Hypothetical view of the interaction of bacteriophages with mammalian immune cells. Phages are able to interact with (currently unknown) immune receptors and induce corresponding immune responses. The immune responses induced by the bacteriophages can either be pro- of anti-inflammatory. For instance, the tested phages are able to induce the pro-inflammatory cytokines IL1α and IL1β. Through the induction of IL1RN by the phage, the phage is able to inhibit the pro-inflammatory responses that are otherwise induced by these cytokines. Pro-inflammatory cytokines are marked in red (*i*.*e*. TNF-α, IFN-γ,IL1α, IL1β, IL-6, IL-17, CXCL1 and CXCL5), anti-inflammatory cytokines are depicted in green (*i*.*e*. TGF-β, IL-4, IL-10 and IL1RN). Dark orange depicts proteins that play a key role in the removal or perception of bacterial pathogens (*i*.*e*. CD14 and LYZ).

At this point it is difficult to establish whether the phages are predominantly pro- or anti-inflammatory, although the phages have the tendency to induce an anti-inflammatory response, as assessed on the basis of these twelve immunity-related genes and on the premise of a sufficiently high phage concentration (*i*.*e*. 10^3^ pfu/PBMC). It is on the other hand very clear that they do activate several immunological pathways (Fig. 6).

The influence of phages on *e*.*g*. cytokine mRNA production, as observed in this study, may not be all that unexpected, since there are several indications for other interactions of phages with human cells. For example, they may play a significant role in transplantation as it was shown that in mice they reduce cellular infiltration of allogenic skin allografts^44^. Phages might do this by inhibiting the adhesion of platelets and, to some extent, of T- cells to fibrinogen, a protein which plays an important role in transplant rejection, angiogenesis and metastasis^45, 46^. A more recent study demonstrated that bacteriophages are also able to inhibit the production of reactive oxygen species (ROS) as part of the response of granulocytes to the presence of bacteria, which may be beneficial because overproduction of ROS leads to tissue damage^47, 48^. Another study proved that phages can aid in the killing of phagocytosed *S*. *aureus* ^49^. The authors showed that phage particles absorbed to the bacterial surface have an impact on the killing of engulfed *S*. *aureus* inside phagocytic cells (*e*.*g*. macrophages) by 38.7%. Furthermore, it has been indicated that phages are able to interact with human mucosal surfaces and form a non-host derived immune barrier^50, 51^.

It was recently shown that the surface proteins gp23*, gp24*, Hoc and Soc of phage T4 do not induce an *IL10* response, neither as elements of the integral phage capsid nor as isolated proteins. This is in accordance to our findings for *S*. *aureus* phage ISP, but not for the *P*. *aeruginosa* phages^18, 52^. Nor did these authors detect the presence of pro-inflammatory cytokines such as TNFA, IL1 and IL6 in mice, murine dendritic cell cultures or human blood. Contrary to what we show here, they postulate (based on their findings) that bacteriophages do not induce an immune response. This difference in results might be explained by the use of different incubation times, since we used 20 h as a stimulation time (optimal time frame for the detection of *IL10* mRNA) instead of 5–6 h. Another major difference is that they studied an *Escherichia coli* phage (T4), which might suggest a species/phage specific response.

The up-regulation of *IL1RN* by all five phages, as shown in our study, is of particular interest. All five phages induce the pro-inflammatory cytokines IL1A and IL1B, but the simultaneous induction of the anti-inflammatory IL1 receptor antagonist (IL1RN) will interfere with the pro-inflammatory IL1 signal and thus dampen the IL1 pro-inflammatory response.

In a previous study we showed the difficulty to remove endotoxins from phage preparations^53^, making it impossible to completely remove all endotoxins from high titer *P*. *aeruginosa* phage preparations. To exclude that the observed immune response for the *P*. *aeruginosa* phages were, partially, due to incomplete endotoxin removal, we compared the response of *S*. *aureus* phage ISP, shown to be completely endotoxin-free with that of ISP phage in combination with three concentrations of endotoxins (*i*.*e*. 0.1, 10^−3^ or 10^−7^ EU/PBMC). Our results strongly indicate that the addition of even the highest concentration of endotoxins to a high concentration of *S*. *aureus* phage ISP (*i*.*e*., 10^3^ pfu/PBMC) has no effect on the induced immune response (Figs 2A and S1). Intriguingly, when an intermediate level of endotoxins was added, the immune response tipped to a more anti-inflammatory response (*i*.*e*. an up-regulation of IL1RN and a strongly reduced expression of CXCL1 and CXCL5) and differed from that after stimulation with the highly purified phage or with only endotoxin.

## Conclusions

The up-regulation of *IL1RN* (Interleukin-1 Receptor Antagonist) and *SOCS3* (Suppressor of Cytokine Signaling 3) by all five phages, or the down-regulation of *CXCL1* and *CXCL5* by *S*. *aureus* phage ISP in addition of endotoxins, might indicate that these phages have evolved anti-inflammatory properties while some pro-inflammatory properties remain (*e*.*g*. up-regulation of *IL1A*, *IL1B* and *TNFA*). It could thus be postulated that by reducing the immune response, phages first reduce the chance to be removed from the human body and be degraded. Secondly, these responses might promote the survival of their bacterial host which in turn provides the phage with a higher fitness and better opportunities to proliferate and remove the pathogenic bacteria, in case of bacterial infections. With regard to phage therapy, these anti-inflammatory effects of phages might increase the capacity of the phages to suppress bacterial numbers and thus infections, with the addition of dampening the negative side of the inflammatory response.

## Material and Methods

### Culture of *Pseudomonas aeruginosa* and *Staphylococcus aureus* strains

*Pseudomonas aeruginosa* strain 573 (received from the Eliava IBMV, Tbilisi, Georgia) and *Staphylococcus aureus* strain ATCC 6538 were grown on a Lysogeny Broth (LB) agar plates (Becton Dickinson, Erembodegem, Belgium) and incubated overnight at 37 °C. One colony was subsequently used to inoculate a 15 ml tube containing a 4 ml LB agar slant, and incubated again overnight at 37 °C. Five ml saline was added to yield a suspension with a final concentration of 10^7^ cfu/ml, as confirmed by culture of serial tenfold dilutions.

### Bacteriophage preparation

#### Phage propagation

Bacteriophage stocks (Table 1) were prepared using the double-agar overlay method as described in Merabishvili *et al*.^54^. Briefly, one ml of phage preparation containing 10^6^ plaque forming units (pfu) of bacteriophages was mixed with 3 ml of molten (45 °C) LB top Bacto agar (0.6%) (Becton Dickinson) and 100 µl of the host strain suspension (end concentration of 10^7^ cfu/ml) in a sterile 14 ml tube (Falcon, Becton Dickinson). This mixture was plated onto freshly made 90 mm diameter Petri dishes (Plastiques Gosselin, Menen, Belgium), filled with a bottom layer (20 ml) of 1.5% LB agar, and incubated aerobically at 32 °C for 16 h. Subsequently, 200 µl of chloroform was added to the lids of the Petri dishes and the inverted plates were further incubated at 4 °C for 1 h. The top layer of the double-agar layer was scraped off using a sterile Drigalski spatulum and transferred to a sterile 50 ml tube.

The harvested phages were centrifuged for 20 min at 6,000 × *g* at 4 °C. The supernatant was aspirated using a sterile 10 ml syringe (BD Plastipak, Becton Dickinson) with a 30G sterile needle (BD Microlance 3, Becton Dickinson) and passed through a 0.22 µm membrane filter (Sartorius Stedim, Zellik, Belgium). The filtrate was subsequently centrifuged at 35,000 × *g* for one hour. The phage pellet was resuspended in 5 ml saline and stored at 4 °C overnight before determining the phage titer. Preferably, the titer of the phage lysate should be checked at least one day later according to the above described procedures. This will allow phage particles that may have clumped together during centrifugation steps to disengage^21^.

#### Phage titer determination

The bacteriophage titer was determined by assaying decinormal serial dilutions (log(0) to log(−12)) of the bacteriophage suspension with the overlay method^54^. One ml of each dilution was mixed with 3 ml of molten (45 °C) LB 0.6% top Bacto agar and the host strain (end concentration of 10^7^ cfu/ml) in a sterile 14 ml tube. This mixture was plated in triplicate onto 90 mm diameter Petri dishes, filled with a bottom layer of 1.5% LB agar, and incubated for 16 h at 37 °C. To determine the original bacteriophage concentration, plates with one to 100 distinguishable plaques were counted. The mean was then calculated for the triplicate plates.

#### Endotoxin removal

Endotoxins were removed and quantified as described in Van Belleghem *et al*.^53^. Briefly, phage lysates were further purified using CsCl centrifugation. Subsequently, endotoxin concentrations were determined using the EndoZyme recombinant Factor C (rFC) Assay (Hyglos, Bernried am Starnberger See, Germany). For the *P*. *aeruginosa* phages, 10^−5^–10^−10^ EU/pfu remained after the CsCl purification. The *S*. *aureus* phage ISP contained no endotoxins (Table 1).

### PBMC isolation

Peripheral Blood Mononuclear Cells (PBMCs) were isolated from a buffycoat after informed consent (Blood Transfusion Centre, Ghent), using a Lymphoprep (Axis-Shield, Dundee, Scotland) gradient. Fifty ml of the buffycoat was added to 250 ml Hank’s Balanced Salt Solution, without Ca^2+^ and Mg^2+^ (HBSS) (Invitrogen). Of this dilution, eight aliquots of 35 ml were each added to 15 ml Lymphoprep in a 50 ml Falcon tube. These mixtures were subsequently centrifuged at 500 x *g* for 20 min at room temperature. The inner whitish ring of PBMCs, present between the lymphoprep and the plasma phase, was transferred to 25 ml HBSS and centrifuged at 450 × *g* for 10 min at room temperature. The supernatants was removed and the cell pellet was resuspended in 10 ml HBSS. All resuspended cells were pooled into a 50 ml Falcon tube and HBSS was added to a total volume of 50 ml. A small fraction of this cell solution was used to count the number of cells present, before it was centrifuged again at 350 × *g* for 10 min at room temperature.

The total number of cells was counted using a Sysmex KX-21 (Sysmex, Norderstedt, Germany). The cell pellet was resuspended in heat-inactivated foetal calf serum with 10% dimethyl sulfoxide (DMSO) to a concentration of 2 × 10^7^ cells/ml and divided in 1 ml aliquots before cryostorage them in liquid nitrogen.

### Stimulation of PBMCs

Stimulations were performed in 100 µl volumes containing 10^6^ PBMCs. One vial of stored PBMCs, containing 2 × 10^7^ cells/ml, was thawed at 37 °C prior to adding 9 ml HBSS (without Ca^2+^ and Mg^2+^). This suspension was subsequently centrifuged at 350 × *g* for 10 min. The obtained cell pellet was resuspended in 5 ml HBSS (without Ca^2+^ and Mg^2+^) and 80 µl was used for cell counting on a Sysmex KX-21. This cell suspension was centrifuged at 350 × *g* for 10 min. The resulting cell pellet was resuspended in RPMI 1640 (supplemented with 2 mM L-glutamic acid, 1X MEM non-essential amino acids, 1 mM sodium pyruvate, 60 U of penicillin/ml, 10 mg/ml streptomycin, 2 mM L-glutamine and 10% heat inactivated foetal calf serum) to a final concentration of 10^7^ cells/ml^55^.

The PBMCs (*i*.*e*. 10^6^ PBMCs/100 µl) were subsequently stimulated with 10 µl of the bacterial host (*i*.*e*. either *P*. *aeruginosa* or *S*. *aureus*, at a concentration of 10^7^ cfu/ml, *i*.*e*., 10^5^ cfu per 10^6^ PBMC or 0.1 cfu/PBMC), 10 µl of the phage suspensions (*i*.*e*. either a phage lysate or a highly purified (CsCl) phage suspension (Table 1)) at concentrations between 10^13^ and 10^9^ pfu/ml, *i*.*e*., 10^5^ or 10 pfu/PBMC, or phage suspension in combination with 10 µl endotoxins (commercial preparation derived from *P*. *aeruginosa* strain 10; Sigma Aldrich) at concentrations of 10^1^, 10^5^ or 10^7^ Endotoxin units/ml (EU/ml), *i*.*e*., 0.1, 10^−3^ or 10^−7^ EU/PBMC. As a negative control, 10 µl saline was added to the cells. The PBMCs were incubated for 20 h at 37 °C in 5% CO_2_. All stimulation experiments and controls were carried out on six biological replicates (*i*.*e*. anonymous donors) in triplicate. We confirm that all methods were carried out in accordance to relevant guidelines and regulations and that all experimental protocols were approved by the ethical committee of the university of Ghent (EC/2017/0558).

#### Extraction of total nucleic acids

After 20 h of PBMC stimulation, the total cell volume was transferred to 1 ml Qiazol (Qiagen, Valencia, CA) and stored at −80 °C for at least 16 h before extracting the RNA. The total RNA fraction was isolated using a semi-automated procedure of NucliSens EasyMag (Biomérieux, Marcy l’Étoile, France). Briefly, 900 µl EasyMag lysis buffer was added to the 1.1 ml Qiazol solution. This mixture was subsequently transferred to an NucliSens EasyMag cartridge. Finally, 100 µl magnetized silica was added and the cartridge was loaded on the machine (according to the manufacturer). In a final step, the RNA was eluted with 35 µl NucliSens EasyMag elution buffer.

#### DNase digest

DNase digest was performed immediately after nucleic acid extraction to remove DNA from the sample. Five µl of the nucleic extract was added to one µl of DNase (1 U/µl), one µl 10X DNase reaction buffer (Promega, Mannheim, Germany) and three µl of RNase free H_2_O to make up a total volume of 10 µl. This mixture was incubated for 30 min at 37 °C. The DNase digestion was terminated by adding 1 µl of DNase Stop Solution and incubating the mixture at 65 °C for 10 min. DNase digested RNA samples were stored at −80 °C.

#### cDNA Library preparation and sequencing

*For transcriptome analysis purposes:* cDNA preparation and Illumina HiSeq^TM^ sequencing was performed at the Beijing Genome Institute (BGI) as described by Ren *et al*.^56^. The cDNA libraries were prepared according to the manufacturer’s instructions (Illumina, San Diego, CA). Beads coated with oligo(dT) were used to isolate eukaryotic poly(A) mRNA from the total RNA. Purified mRNA was then fragmented in RNA fragmentation buffer (Ambion, Austin, TX). Using these short fragments as templates, random hexamer-primers (Illumina) were used to synthesize the first-strand cDNA. The second-strand cDNA was synthesized using buffer, dNTPs, RNase H and DNA polymerase I. Short double-stranded cDNA fragments were purified with a QIAquick PCR extraction kit (Qiagen) and eluted with elution buffer for end repair and the addition of a terminal ‘A’ nucleotide. Next, Illumina sequencing adaptors were ligated to the DNA fragments. DNA fragments of a selected size were gel-purified and amplified by PCR. The amplified library was sequenced on an Illumina HiSeq^TM^ 2000 sequencing machine at BGI, using single-end sequencing with an expected library size of 160 bp and a read length of 90 nt.

*For RT-qPCR purposes*: The cDNA synthesis was preformed using the RevertAid RT kit (Thermo Scientific, Waltham, MA) in four times 40 µl volumes according to the manufacturer’s instructions.

#### Raw read filtering, mapping expression quantification

The images generated by the sequencer were converted into nucleotide sequences by a base-calling pipeline. The raw reads were saved in fastq format, and the dirty raw reads were removed prior to analyzing the data. Dirty raw reads were removed according to the following criteria: reads with sequence adaptors, reads with more than 10% ‘N’ bases; low quality reads (*i*.*e*. the percentage of low quality bases is over 50% in a read, BGI defined the low quality base to be the base whose sequencing quality is no more than 10). All subsequent analyses were based on clean reads.

The reference sequences used were human genome and transcriptome sequences downloaded from the UCSC website (version hg19). Clean reads were respectively aligned to the reference genome and transcriptome using SOAP2^57^. No more than three mismatches were allowed in the alignment for each read.

Reads that could be uniquely mapped to a gene were used to calculate the expression level. The gene expression level was measured by the number of uniquely mapped reads per kilobase of exon region per million mappable reads (RPKM)^58^.

#### Differentially expressed gene (DEG) analysis

Using ‘The significance of digital gene expression profiles’^59^, differentially expressed genes between *P*. *aeruginosa*-stimulated PBMCs and non-stimulated PBMCs were identified, based on the following criteria: False Discovery Rate (FDR) ≤0.001 and an absolute fold change ≥2. The data discussed in this publication have been deposited in NCBI’s Gene Expression Omnibus^60^ and are accessible through GEO Series accession number GSE95573 (https://www.ncbi.nlm.nih.gov/geo/query/acc.cgi?acc=GSE95573).

#### Expression pattern analysis of DEGs

Based on the assumption that genes that have a similar expression pattern usually have a functional correlation, cluster analysis of gene expression patterns was performed using the *cluster* ^61^ and *javaTreeview* ^62^ software.

#### Gene ontology (GO) analysis of DEGs

The gene ontology analysis of DEGs was performed by a GO enrichment analysis, which provides all GO terms that are significantly enriched in a list of DEGs by comparing to a genome background and subsequent filtering the DEGs that correspond to specific biological functions. In this method all DEGs are first mapped to GO terms in the GO database (http://www.geneontology.org), calculating gene numbers for every term, then using hypergeometic tests to find significantly enriched GO terms in the input list of DEGs, based on ‘GE::TermFinder’ (http://smd.stanford.edu/help/GO-TermFinder/GO_TermFinder_help.shtml).

### Gene expression analysis of 12 immunity-related genes

#### RT-qPCR

Based on the RNA seq data, twelve target genes were selected for further evaluation by RT-qPCR. Combining the datasets of both the phage PNM lysate and *P*. *aeruginosa* stimulated PBMCs, we observed that a total of 2679 genes were uniquely expressed. As a cut-off for qPCR detection, a minimum of 7000 reads was selected either in the control condition or after stimulation, to ensure detection in the RT-qPCR. This led to a reduction to 418 genes. Furthermore, to detect a difference in gene expression in the qPCR, a Log2Fold change difference was needed after normalization of the target genes by the reference gene *ACTB*. This led to a further reduction to 176 potential target genes for qPCR-based analysis. From these, a total of twelve genes were selected on the basis of their possible importance in the immune response, *i*.*e CD14*, *CXCL1*, *CXCL5*, *IL1A*, *IL1B*, *IL1RN*, *IL6*, *IL10*, *LYZ*, *SOCS3*, *TGFBI* and *TNFA* (Table S6).

The RT-qPCR was performed in a total reaction volume of 10 µl using 0.5 µM of each primer (Table S5), 2.5 µl cDNA and LightCycler 480 High resolution Melting master (Roche Applied Sciences, Indianapolis, Indiana).

The selection of the most stable reference gene(s) was carried out as described by Vandesompele *et al*.^63^ using SybrGreen as means of detection. Using geNorm, we found that all six reference genes (*i*.*e*. *ACTB*, *GADPH*, *HRPT1*, *PPIA*, *TBP and UBC*; Table S7) are sufficiently stable (internal control gene-stability measure M < 1.5). Therefore, the minimal optimal number of reference targets to be used in this experiment was 1 (V < 0.15).

The mRNA levels are expressed in relative copy numbers normalized against the reference gene (ACTB), as described by Giulietti *et al*.^64^ and Samarasinghe *et al*.^65^.

### Importance

We show that bacteriophages are able to induce an (innate) immune response, in contrast to some other recent reports. Additionally, we have strong indications that the phage-induced response is overall anti-inflammatory, which may contribute to the beneficial therapeutic effects of phages during phage therapy.

## Electronic supplementary material

Supplementary data

Supplementary Table S2

Supplementary Table S3

Supplementary Table S4
