# Supplementary material for: Pro- and anti-inflammatory responses of peripheral blood mononuclear cells induced by Staphylococcus aureus and Pseudomonas aeruginosa phages
Source: Sci Rep. 2017 Aug 14;7:8004. doi: 10.1038/s41598-017-08336-9 (PMC5556114; doi:10.1038/s41598-017-08336-9)
Supplement: Supplementary file 1 — Supplementary data [file 41598_2017_8336_MOESM1_ESM.pdf]

Pro- and anti-inflammatory responses of peripheral blood mononuclear cells induced by *Staphylococcus aureus* and *Pseudomonas aeruginosa* phages

Jonas Van Belleghem[1], Frédéric Clement[2], Maya Merabishvili[3, 1], Rob Lavigne[4], Mario Vaneechoutte[1]

[1]Laboratory Bacteriology Research, Department of Clinical Chemistry, Microbiology and Immunology, University Ghent, Medical Research Building II, De Pintelaan 185, 9000, Ghent, Belgium.

[2] Center for Vaccinology, Ghent University Hospital, Ghent, Belgium

[3] Laboratory for Molecular and Cellular Technology (LabMCT) Queen Astrid Military Hospital, Bruynstraat 1, 1120 Brussels, Belgium

[4] Laboratory of Gene Technology, KULeuven, Kasteelpark Arenberg 21 box 2462, 3001, Leuven, Belgium.

**Corresponding author: van.belleghem.jonas@gmail.com*

Address: Laboratory Bacteriology Research, Medical Research Building II, Ghent University Hospital, De Pintelaan 185, 9000 Gent, Belgium

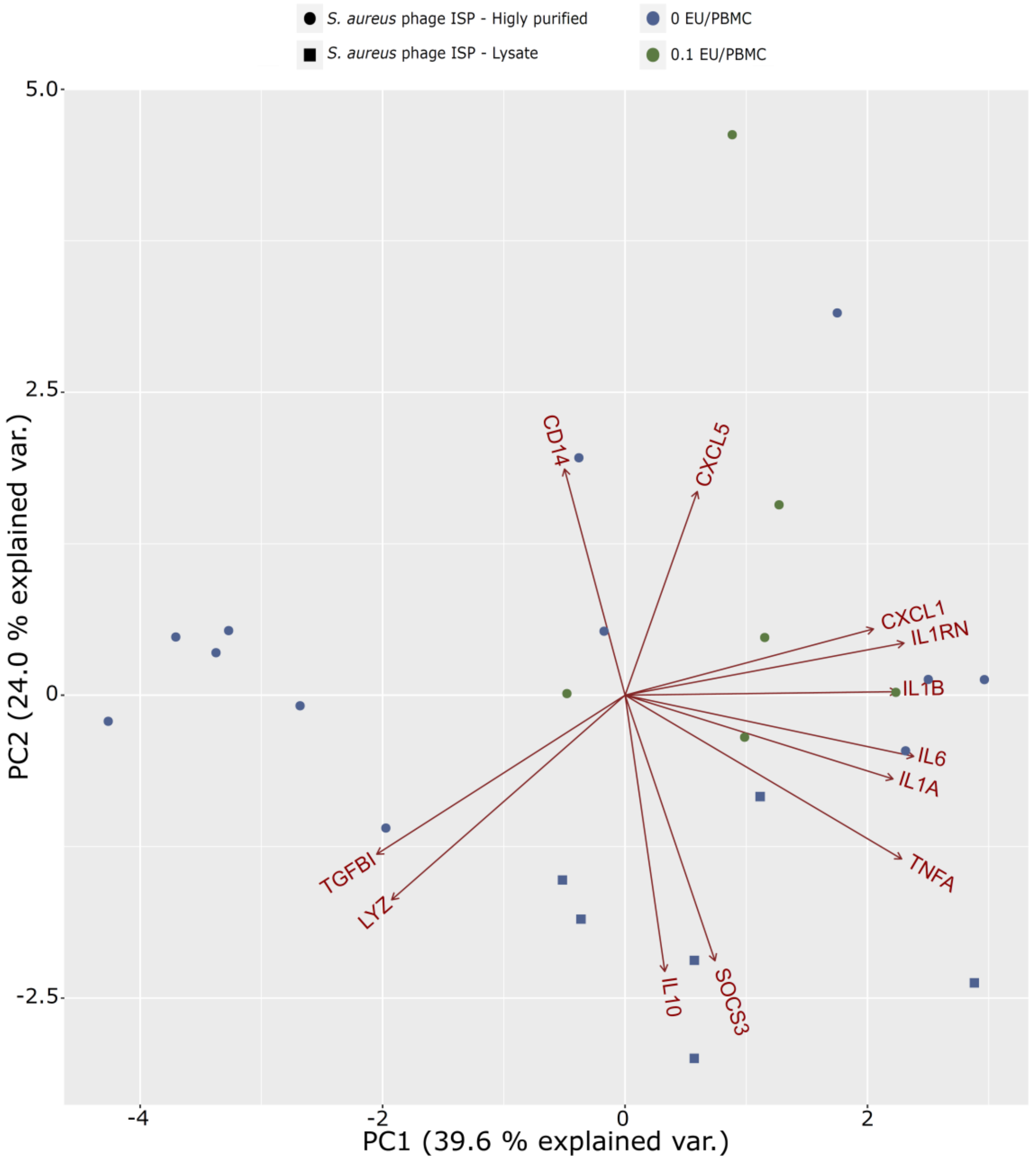

Figure S 1: Principal components analysis of *S. aureus* phage ISP with or without the addition of 10^{-1} EU/PBMC. The immune response induced by the highly purified phage ISP (●) differs from the one induced by the phage ISP lysate (■), as these two groups are visibly separated. When endotoxins are added to a final concentration of 10^{-1} EU/PBMC is added to the highly purified phage (●), the response is similar to the highly purified phage (●) and not the phage lysate (■), indicating that the observed difference is not due to the presence of LPS but due to bacterial proteins present in the phage lysate.

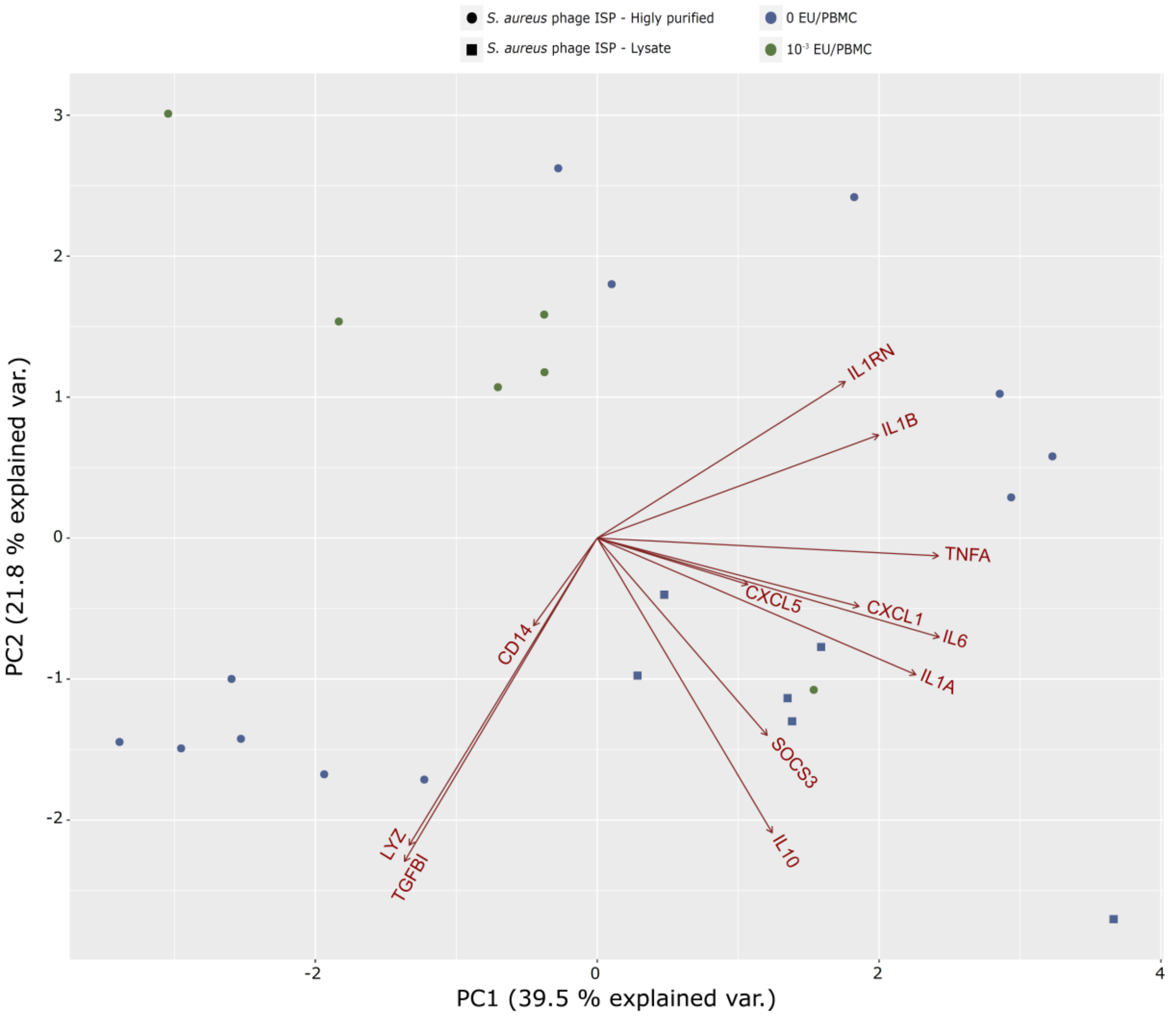

Figure S 2: Principal components analysis of *S. aureus* phage ISP with or without the addition of 10^{-3} EU/PBMC. The immune response induced by the highly purified phage ISP (●) differs from the one induced by the phage ISP lysate (■), as these two groups are visibly separated. When endotoxins are added to a final concentration of 10^{-3} EU/PBMC is added to the highly purified phage (●), the response is similar to the highly purified phage (●) and not the phage lysate (■), indicating that the observed difference is not due to the presence of LPS but due to bacterial proteins present in the phage lysate.

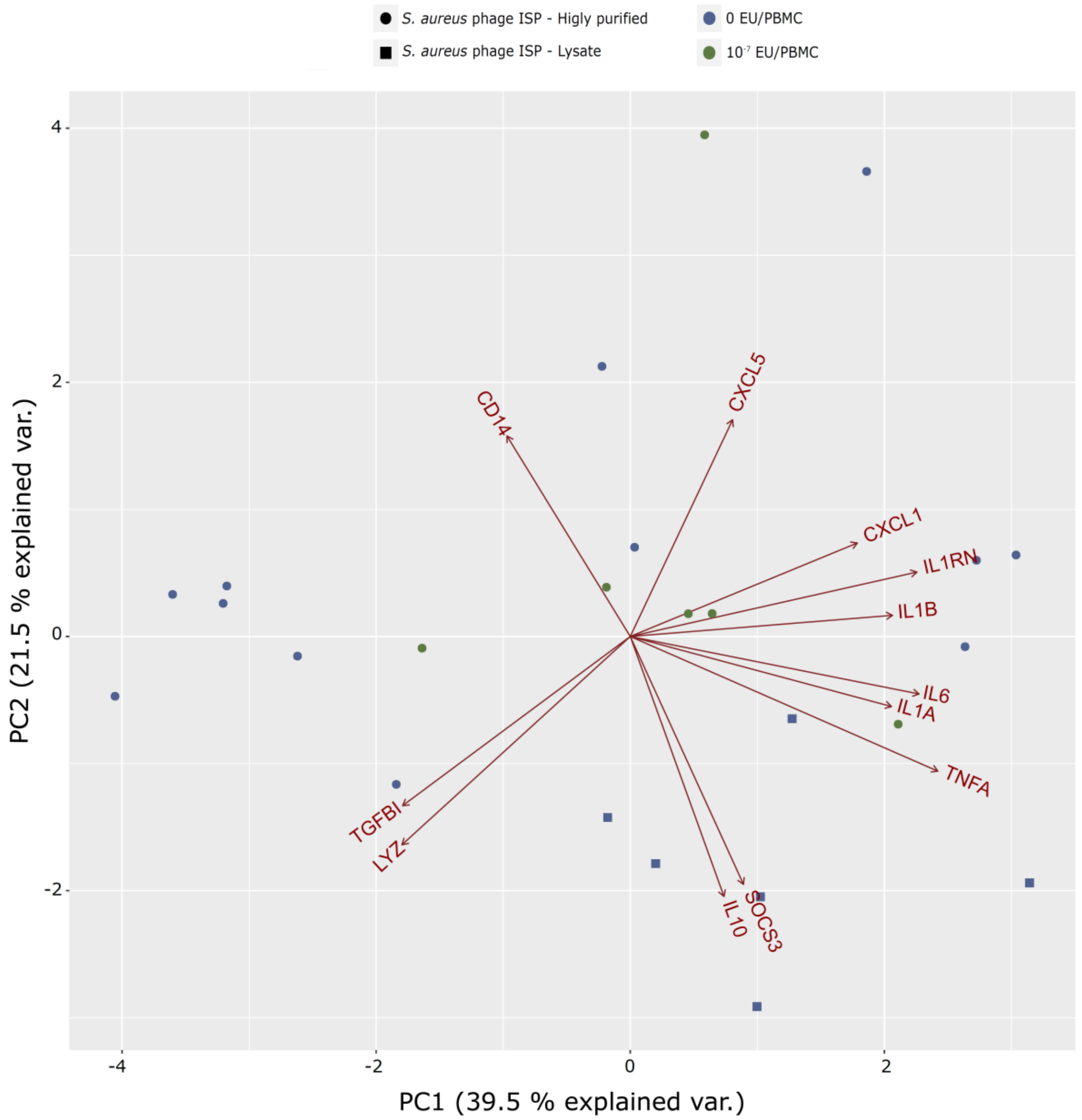

Figure S 3: Principal components analysis of *S. aureus* phage ISP with or without the addition of 10^{-7} EU/PBMC. The immune response induced by the highly purified phage ISP (●) differs from the one induced by the phage ISP lysate (▪), as these two groups are visibly separated. When endotoxins are added to a final concentration of 10^{-7} EU/PBMC is added to the highly purified phage (●), the response is similar to the highly purified phage (●) and not the phage lysate (▪), indicating that the observed difference is not due to the presence of LPS but due to bacterial proteins present in the phage lysate.

Table S1: Transcriptome summary of human PBMCs either not stimulated or stimulated with *P. aeruginosa* or *P. aeruginosa* phage PNM.

Stimulation condition	Unstimulated PBMCs			PBMCs stimulated with phage PNM (10^3 pfu/PBMC)			PBMCs stimulated with *P. aeruginosa* PA573 (10^{-1} cfu/PBMC)		
Biological replicate	a	b	c	a	b	c	a	b	c
Clean reads	46,853,888	46,795,416	47,094,320	47,611,412	47,639,850	47,629,112	47,302,700	48,948,918	47,331,732
Genome map Rate (%)	86.30%	85.80%	85.60%	85.50%	85.60%	85.30%	85.50%	86.10%	85.30%
Gene map Rate (%)	79.30%	79.50%	81.30%	81.00%	80.60%	80.90%	79.20%	78.00%	78.00%
Expressed Genes	16,94	16,968	16,812	16,867	16,885	16,975	17,025	17,099	17,001
Novel Transcripts	644	673	592	683	733	698	746	838	778
Alternative Splicing	55,961	54,175	48,512	59,912	56,444	57,258	58738	60153	59122
SNP	51,195	49,806	46,9	50,326	51,246	51,141	52,877	56,391	54,279

Table S5: List of primers used in the RT-qPCR for the validation of the RNA seq. The F denotes the forward primer and the R denotes the reverse primer.

Names	Sequence (5' - 3')	Tm (°C)
IL10_F	CATCGATTTCTTCCCTGTGAA	45.3
IL10_R	TCTTGGAGCTTATTAAAGGCATTC	47.2
TNFa_F	CCCAGGGACCTCTCTCTAATC	51.2
TNFa_R	ATGGGCTACAGGCTTGTCACT	49.2
ACTB_F	GGATGCAGAAGGAGATCACTG	49.2
ACTB_R	CGATCCACACGGAGTACTTG	62.0
SOCS3_Fw	GGCCACTCTTCAGCATCTC	60.0
SOCS3_Rv	ATCGTACTGGTCCAGGAACTC	49.2
TGFBI_F	GAAGGGAGACAATCGCTTTAGC	49.7
TGFBI_R	TGTAGACTCCTTCCCGGTTGAG	51.6
CD14_F	CGCTCCGAGATGCATGTG	58
CD14_R	TTGGCTGGCAGTCCTTTAGG	62
LYZ_F	AAAACCCCAGGAGCAGTTAAT	45.3
LYZ_R	CAACCCTCTTTGCACAAGCT	60
CXCL5_F	ATCTGCAAGTGTTCGCCATAG	47.3
CXCL5_R	ACAAATTTCCTTCCCGTTCTTC	46
IL1B_F	GGCCACATTTGGTTCTAAGAAA	46
IL1B_R	TAAATAGGGAAGCGGTTGCTC	47.3
IL1RN_F	GAAGATGTGCCTGTCCTGTGT	49.2
IL1RN_R	CGCTCAGGTCAGTGATGTTAA	47.3
IL1A_F	CGCCAATGACTCAGAGGAAGA	49.2
IL1A_R	AGGGCGTCATTCAGGATGAA	60
IL6_F	GGTACATCCTCGACGGCATC	64
IL6_R	GCCTCTTTGCTGCTTTCACAC	49.2
CXCL1_F	GGAAAGAGAGACACAGCTGCA	49.2
CXCL1_R	AGAAGACTTCTCCTAAGCGATGC	50.2

Table S6: Selection of the 12 target genes for further analysis by RT-qPCR. Selection was based on number of reads in either control of stimulated condition (*i.e* . phage PNM lysate or *P. aeruginosa* strain PA573) which needs to exceed 6000 and the Log2ratio which needs to be larger than |2|. NA indicated no differential expression for a specific condition/gene, hence no number reads were included.

Symbol	# reads control	# reads phage PNM lysate	# reads *P. aeruginosa* strain PA573	Log2Ratio phage PNM lysate	Log2Ratio *P. aeruginosa* strain PA573
TGFBI	34300	640	62	-5.68	-9.27
CD14	134382	NA	594	NA	-7.96
LYZ	42484	3997	206	-3.34	-7.83
CXCL5	249901	NA	8756	NA	-4.97
IL1B	30961	636519	137021	4.44	2.01
IL1RN	3167	41322	19681	3.78	2.5
TNF	2260	4823	16990	1.17	2.77
IL1A	418	28048	8740	6.15	4.25
IL10	363	6890	7771	4.32	4.28
IL6	573	51002	52356	6.55	6.37
SOCS3	20597	79658	NA	2.02	NA
CXCL1	20185	152399	NA	2.99	NA

Table S7: Primers used to determine the most stable reference genes. The F denotes the forward primer and the R denotes the reverse primer.

mRNA target	Name primer	Sequence (5’- 3’)
ACTB	ACTB_F	GGATGCAGAAGGAGATCACTG
	ACTB_R	CGATCCACACGGAGTACTTG
HPRT1	HPRT1_F	TCAGGCAGTATAATCCAAAGATGGT
	HPRT1_R	AGTCTGGCTTATATCCAACACTTCG
UBC	UBC_F	TCGCAGCCGGGATTTG
	UBC_R	GCATTGTCAAGTGACGATCACA
TBP	TBP_F	ATGTGAAGTTTCCTATAAGGTTAG
	TBP_R	AGGAAATAACTCTGGCTCATAAC
GAPDH	GAPDH_F	TCACCACCATGGAGAAGGC
	GAPDH_R	GCTAAGCAGTTGGTGGTGCA
